# Spatial patterns and influencing factors of financial agglomeration in Guangdong-Hong Kong-Macao Greater Bay Area

**DOI:** 10.1371/journal.pone.0306301

**Published:** 2024-08-01

**Authors:** Yujun Wei, Mengbin Wang, Xiaokun Wei, Fan Yuan, Jie Fan, Shusong Ba

**Affiliations:** 1 Development Strategy and Cooperation Center, Zhejiang Lab, Hangzhou, China; 2 Zhejiang Laboratory of Philosophy and Social Sciences -Laboratory of Intelligent Society and Governance, Zhejiang Lab, Hangzhou, China; 3 School of Optoelectronic Science and Engineering, University of Electronic Science and Technology of China, Chengdu, China; 4 Department of Applied Mathematics and Computer Science, Technical University of Denmark, Lyngby, Denmark; 5 Institute of Geographic Sciences and Natural Resources Research, Chinese Academy of Sciences, Beijing, China; 6 Peking University HSBC Business School, Peking University, Shenzhen, China; AUM: American University of the Middle East, KUWAIT

## Abstract

The Guangdong-Hong Kong-Macao Greater Bay Area (GBA) represents a significant economic zone with a diverse financial landscape. Understanding the spatial distribution of financial resources within this area is crucial for promoting balanced economic growth and financial development. This study investigates the spatial patterns of financial agglomeration in the GBA, identifying key influencing factors and assessing their impact on the region’s financial landscape. We employ the entropy value method to evaluate financial agglomeration levels across the GBA’s cities. Additionally, we use spatial econometric techniques to analyze the spatial correlations and the Geo-Detector model to determine the primary factors influencing financial agglomeration. The analysis reveals an overall increase in financial agglomeration, with significant disparities among cities. Key factors driving this agglomeration include transportation infrastructure, overseas trade, foreign direct investment (FDI), and technological advancements. Hong Kong and Shenzhen display notable unevenness in the distribution of financial industries. The interplay between finance, technology, and industrial sectors suggests considerable development potential. Understanding and optimizing the spatial distribution of financial resources is essential for fostering high-quality financial development and sustainable economic growth in the GBA. This study provides insights that can inform policy decisions aimed at enhancing financial integration and cooperation within the region.

## 1.Introduction

The evolution of mature global bay areas highlights the crucial role finance plays as a catalyst for transformational development [1]. Financial hubs like New York, Tokyo, and San Francisco operate under paradigms combining finance with services, industry, and technology, respectively. This underscores the pivotal role of the financial sector in driving regional growth [2]. At its core, finance facilitates capital mobilization, bridging value across time and space. Financial centers, as resource allocation hubs, effectively channel various "flows" (human capital, logistics, and finances), benefiting from economies of scale and agglomeration [3]. Drawing parallels with these mature bay areas, finance is poised to be the key in the GBA’s future growth trajectory. Beyond its traditional economic impetus, financial agglomeration enhances regional innovation and industrial rejuvenation. Concentrated financial entities can leverage shared infrastructural assets, improving operational efficiency. Their spatial proximity facilitates seamless information exchange, bolstering the credibility of both formal and informal data streams [4]. Such financial congregation fosters competition, inducing financial entities to innovate and introduce avant-garde financial products, broadening support for regional innovators and ventures. The resultant effect is an optimized allocation of regional resources, driving the successful fruition of diverse investment initiative [5, 6].

The GBA boasts a comprehensive industrial system. Predominantly, the manufacturing industry, anchored around a high-tech cluster led by Shenzhen, synergizes with modern service hubs in Hong Kong and Macao [7]. In terms of economic magnitude and population, the GBA enjoys a leading edge over other urban agglomerations in China [8]. However, the GBA’s development is characterized by marked disparities, extending beyond economic variances across cities to significant imbalances in the distribution of financial resources [9]. Given finance’s foundational role in the region’s economic framework, it is crucial to continuously bolster the GBA’s long-term trajectory. Therefore, understanding the spatial dynamics of financial agglomeration, its determinants, refining its distribution, and fostering financial sector excellence are pivotal. These efforts are integral to the GBA’s resource reallocation, transformation, and sustained economic growth.

Amid the accelerating trends of economic and financial globalization, the world economy is transitioning from the traditional "center-periphery" paradigm to a more interconnected global network [10]. Within this network, financial transactions can materialize at various nodal points, allowing any region with the requisite conditions to emerge as a financial hub [11]. Empirically, the agglomeration of financial components epitomizes the contemporary financial sector [12]. As technological advancements reshape classical financial theories, the dimension of "space" gains prominence [13]. Correspondingly, the rise of spatial econometrics has prompted scholars to integrate spatial considerations into financial studies, fostering deeper investigations into the geospatial distribution of the financial industry. Contemporary scholarship on the spatial pattern of financial agglomeration has established a foundational research framework. Key areas of inquiry include developing metrics for assessing regional financial agglomeration [14]; spatially identifying regional financial centers [15, 16]; understanding the dynamic evolution of financial agglomeration patterns and their interplay mechanisms [17, 18]; exploring the drivers underpinning regional financial agglomeration [19, 20]; identifying and characterizing the factors influencing financial agglomeration [21–23].

While extensive research has been conducted on the spatial patterns and determinants of financial agglomeration, a significant gap remains in synthesizing these aspects within an integrative empirical framework. Current studies primarily emphasize identifying diverse determinants of financial agglomeration. However, the examination of interrelationships and interactions among these determinants has not been sufficiently explored. Research on the spatial patterns of financial agglomeration predominantly focuses on traditional international financial hubs, notably London and New York [2, 24]. Domestically, studies target provincial and city cluster scales [25] and city cluster scales, highlighting regions such as the Pearl River Delta (PRD), the Yangtze River Delta (YRD), and urban conglomerations like the Beijing-Tianjin-Hebei nexus [17, 26]. While this body of research provides insights into established financial centers and city clusters, it often overlooks the dynamic evolution of financial agglomeration patterns in emerging urban clusters such as the GBA. Key determinants identified in the literature include economic development, human capital, transport accessibility, and governmental support. However, these studies often focus on individual financial metropolises like London, New York [27], Hong Kong [28], Beijing, and Shanghai [29]. This focus on singular cities or metropolitan zones (characterized by uniform regulatory and tariff systems) leaves a significant gap in comprehensive research addressing financial agglomeration patterns in more complex regions like the GBA, with its two systems, three tariff zones, and three currencies.

Our research aims to discern the spatial pattern of financial agglomeration within the "9+2" cities of the GBA, delving into both the determining factors and their interrelations. Specifically, we address: "What constitutes the spatial pattern of financial agglomeration in the GBA’s ’9+2’ cities, and which factors predominantly influence it?" Based on our findings, we offer policy recommendations tailored to the GBA’s prospective developmental trajectories. While past studies extensively explored financial agglomeration, they largely adopted single-factor identification, sidestepping a holistic system-wide perspective examining the interplay among various factors. Recognizing this gap, we employ spatial econometric and geo-detection models to scrutinize the spatial correlations underpinning financial agglomeration. Our approach not only considers individual factors but also emphasizes their synergistic effects. This comprehensive methodology not only strengthens policy analysis but also augments the broader research framework within economic geography.

## 2. Methods

### (1) Study area and temporal scope

Our research focuses on the spatial pattern of financial agglomeration within the "9+2" urban conglomerate of the GBA. This conglomerate includes two special administrative regions (Hong Kong and Macao) and nine cities from Guangdong Province: Guangzhou, Shenzhen, Zhuhai, Foshan, Zhongshan, Dongguan, Huizhou, Jiangmen, and Zhaoqing.

In examining the spatial patterns and influential factors of financial agglomeration in the GBA, this study covers the period from 2005 to 2019. The starting point of 2005 was chosen due to significant improvements in the availability and consistency of financial data, which are essential for conducting a robust spatial econometric analysis. The end point of 2019 was selected for two main reasons:

- Pre-policy Analysis Baseline: Concluding the study in 2019 allows us to analyze the financial agglomeration patterns prior to the issuance of the "Outline Development Plan for the Guangdong-Hong Kong-Macao Greater Bay Area" by the Central Committee of the Communist Party of China and the State Council on February 18, 2019. This provides a critical baseline for assessing the financial landscape before the implementation of new regional coordination and development policies, thus offering valuable insights into the plan’s impact.
- Exclusion of COVID-19 Disruptions: Extending the study period to just before the global disruptions caused by the COVID-19 pandemic ensures that our analysis is not influenced by the extraordinary economic conditions of 2020 and beyond. This approach helps maintain the focus on understanding the long-term spatial patterns and factors influencing financial agglomeration without the confounding effects of the pandemic.

This temporal frame ensures a clear, consistent analysis of the financial agglomeration patterns within the GBA, providing essential context for evaluating the impacts of subsequent regional development policies.

### (2) Spatial analysis of financial agglomeration patterns

We investigate multiple dimensions of financial agglomeration, including the concentration of various financial industries (banking, securities, and insurance), the clustering of financial institutions and the gathering of financial talents (Table 1). To objectively evaluate the extent of financial agglomeration, we employ the entropy weight method. This method provides an unbiased way to assign weights to various indicators, reducing the influence of subjective biases. The computational steps for the entropy weight method are detailed in S1 Text in S1 File.

**Table 1:** Indicators for measuring financial agglomeration in the GBA.

Primary indicators	Secondary indicators	Indicator units	Description
Overall Financial Industry	Value added of the financial industry	Billion yuan	Positive
	Number of legal entities in the financial sector	pcs	Positive
	Number of employees in the financial sector	pcs	Positive
Banking Industry	Balance of deposits in financial institutions	Billion	Positive
	Balance of Loans of Financial Institutions	Billion yuan	Positive
	Number of banks and related financial institutions	pcs	Positive
Insurance industry	Insurance Density	Yuan/person	Positive
	Insurance Depth	%	Positive
Security industry	Asset securitization rate	%	Positive
	Number of listed companies	pcs	Positive
	Number of securities business offices	pcs	Positive

To conduct our spatial analysis, we selected Geoda software due to its robust capabilities in exploring spatial data and visualizing geographic patterns. Geoda is widely recognized for its user-friendly interface and powerful spatial autocorrelation features, which are essential for our analysis of financial agglomeration. This software has been extensively used in economic geography and regional science research, demonstrating its efficacy and reliability in identifying and illustrating spatial relationships and patterns [30]. This approach allows us to assess financial agglomeration levels using natural breakpoints and to visualize these patterns on a map. This enables a more intuitive analysis of the spatial distribution of financial agglomeration in the GBA.

To assess the spatial pattern of financial agglomeration within the GBA, we utilize Spatial Auto Correlation as our primary metric to measure spatial relevance. Spatial Auto Correlation captures whether the financial agglomeration of a given spatial unit aligns with or contrasts against the agglomeration trends of its neighboring units. For a comprehensive analysis, we employ both global *Moran’ s I* and local *Moran’ s I*_*i*_ (also known as *LISA* index):

Global *Moran’ s I*: This index provides a global view of spatial autocorrelation, indicating whether the overall pattern is clustered, dispersed, or random. It is widely used in regional science and economics to validate the presence of spatial clusters or hotspots. By applying *Moran’ s I*, we can determine if financial agglomeration in the GBA exhibits significant spatial dependencies at the macro level [31, 32].

Local Indicators of Spatial Association (*LISA*): While *Moran’ s I* offers a global perspective, *LISA* (local *Moran*’ *s I*_*i*_) provides localized insights by identifying clusters and outliers on a micro scale. *LISA* is particularly useful in revealing hot spots (areas of high financial agglomeration) and cold spots (areas of low financial agglomeration), offering detailed insights into the spatial concentration of financial activities. This localized analysis is crucial for understanding the intricate spatial dynamics within the GBA and has been extensively documented in literature [33].

The combination of these two indices allows for a thorough examination of both global and local patterns of financial agglomeration, ensuring a comprehensive understanding of spatial dependencies. The formulas for Spatial Auto Correlation and Local *Moran’ s I*_*i*_ are provided in S2 Text in S1 File.

### (3) Analyzing the determinants of financial agglomeration

We aim to identify the key determinants of financial agglomeration, such as economic structures, human capital, and infrastructure. Our analytical framework categorizes the determinants into twelve distinct groups, providing a holistic understanding of the multifaceted influences at play. By scrutinizing these determinants, we develop a comprehensive understanding of the dynamics shaping financial agglomeration in the GBA (Table 2). Detailed explanations of the influential factors are available in S3 Text in S1 File.

**Table 2:** Influential factors of financial agglomeration in the GBA.

No.	Detection factors	Index	Unit
X1	Consumption capacity	Per capita retail sales of social consumption	Yuan
X2	Per Capita Output	GDP per capita	Yuan
X3	Industrial Structure	Share of Three Industries	%
X4	FDI Attraction	FDI Inflows	Billion yuan
X5	Overseas Trade	Import and export	Billion yuan
X6	High Education	Higher education per 10,000 people	People per 10,000
X7	Network Communication	Number of cell phones per 10,000 people	pcs per 10,000
X8	Transportation	Average surface freight capacity	Tons/sq km
X9	Technological Output	Patents per 10,000 people	pcs per 10,000
X10	Technological Input	R&D cost to GDP ratio	%
X11	Technological Staff	R&D number per 10,000 people	People per 10,000
X12	Government Capacity	Government finance share	%

To explore these determinants in the GBA, we employed GeoDetector. This tool is effective in quantifying the explanatory power of different factors on spatial phenomena and has been successfully applied in numerous studies to assess the impact of socio-economic and environmental factors on geographical distributions [34]. Its ability to handle complex interactions and sensitivity to spatial heterogeneity make it ideal for our analysis.

GeoDetector allows us to explore the spatial consistency between dependent and independent variables, helping to determine the extent to which independent variables explain dependent variables. The fundamental metric for this relationship is the detection value, represented as *q*. This method provides a quantitative measure of the impact of various factors on the spatial imbalances of financial agglomeration. Understanding these influences aids in informed decision-making and targeted policy interventions. Additionally, understanding interactions offers deeper insights into the complex dynamics between influencing factors, guiding more effective and targeted policy decisions. The specific steps for GeoDetector analysis can be found in S4 Text in S1 File.

### (4) Hypotheses formulation

To frame our analysis within a scientifically robust context, we propose the following hypotheses based on observed dynamics and theoretical foundations relevant to financial agglomeration:

**Hypothesis 1 (H1): Dynamic Evolution of Financial Agglomeration**

We hypothesize that financial agglomeration within the GBA exhibits dynamic evolution from 2005 to 2019. This evolution is influenced by various factors including economic changes, policy developments, and technological advancements. This hypothesis allows for a temporal analysis to understand how financial agglomeration adapts and responds to different stages of regional development and major policy implementations.

**Hypothesis 2 (H2): Differential Impact of Determinants on Financial Agglomeration**

We propose that specific factors such as FDI, transportation infrastructure, and technological advancements have a differential and more significant impact on the development of financial agglomeration within the region. By using the GeoDetector tool, we test this hypothesis quantitatively, assessing the relative importance of these factors in shaping the spatial configuration of financial clusters.

These hypotheses are based on established theories of economic geography and agglomeration, which suggest that spatial economic phenomena are dynamic and influenced by both external and internal factors.

## 3.Result

### (1) Financial agglomeration differences within regions

**Fig 1:**
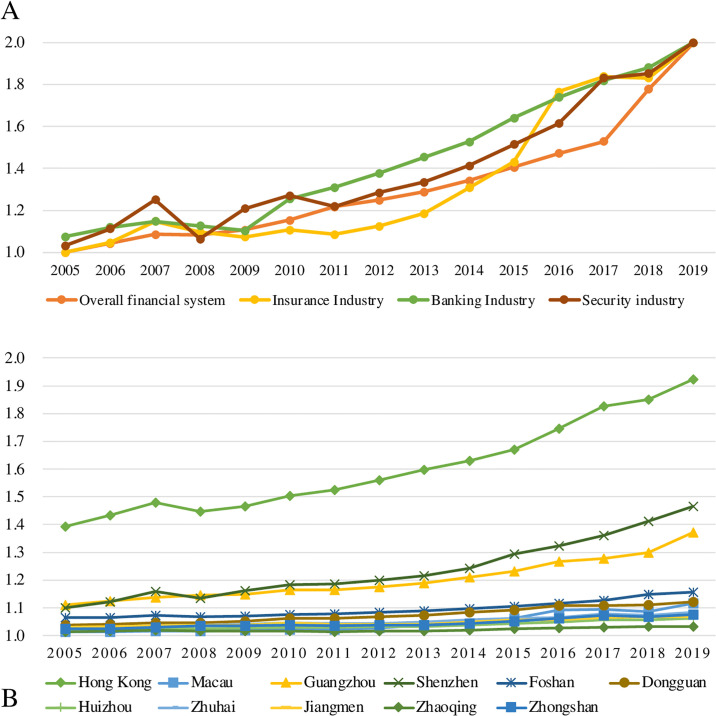
Overview of the evaluation of financial agglomeration in the Guangdong-Hong Kong-Macao Greater Bay Area. (A) Evaluation based on the total size of the financial system, insurance, banking and securities industries; (B) Evaluation of financial agglomeration based on the "9+2" cities.

Our empirical research shows a notable trend in financial agglomeration across the GBA, with significant differences in agglomeration intensities among its cities. The overall financial sectors, including insurance, banking, and securities, exhibit an upward trend in agglomeration (Fig 1A). Specifically, the banking sector has shown a faster pace of agglomeration compared to the insurance and securities sectors. This growth is partly due to government support and increasing demand from businesses and individuals within the GBA. The insurance sector initially lagged in agglomeration but saw a significant increase after 2011, aligning with the banking and securities sectors by 2016. The securities sector experienced a sharp decline in 2008 due to the global financial crisis but recovered steadily from 2011 onwards. The banking and insurance sectors were less affected by the 2008 crisis compared to the securities sector, which is more sensitive to stock and capital market fluctuations.

Within the "9+2" cities of the GBA, there are substantial differences in financial agglomeration intensities (Fig 1B). Hong Kong stands out with a high level of financial agglomeration, despite a notable decline in 2008 due to the global financial crisis. Hong Kong has maintained a consistent upward trend, solidifying its position as a key financial center in the GBA. However, post-2017, the growth in Hong Kong’s financial agglomeration has slowed, potentially due to mainland China’s financial liberalization. Shenzhen has shown remarkable growth in financial agglomeration since 2010, outpacing Guangzhou. Guangzhou, as the provincial capital, has a long history of financial development. Other cities in the GBA, such as Zhongshan, Zhuhai, Jiangmen, and Zhanjiang, have lower financial concentrations compared to Hong Kong, Shenzhen, and Guangzhou. These cities were historically more focused on manufacturing and only recently began developing their financial sectors. Policy and resource allocations favor provincial-level cities, which contributes to the disparities.

**Fig 2:**
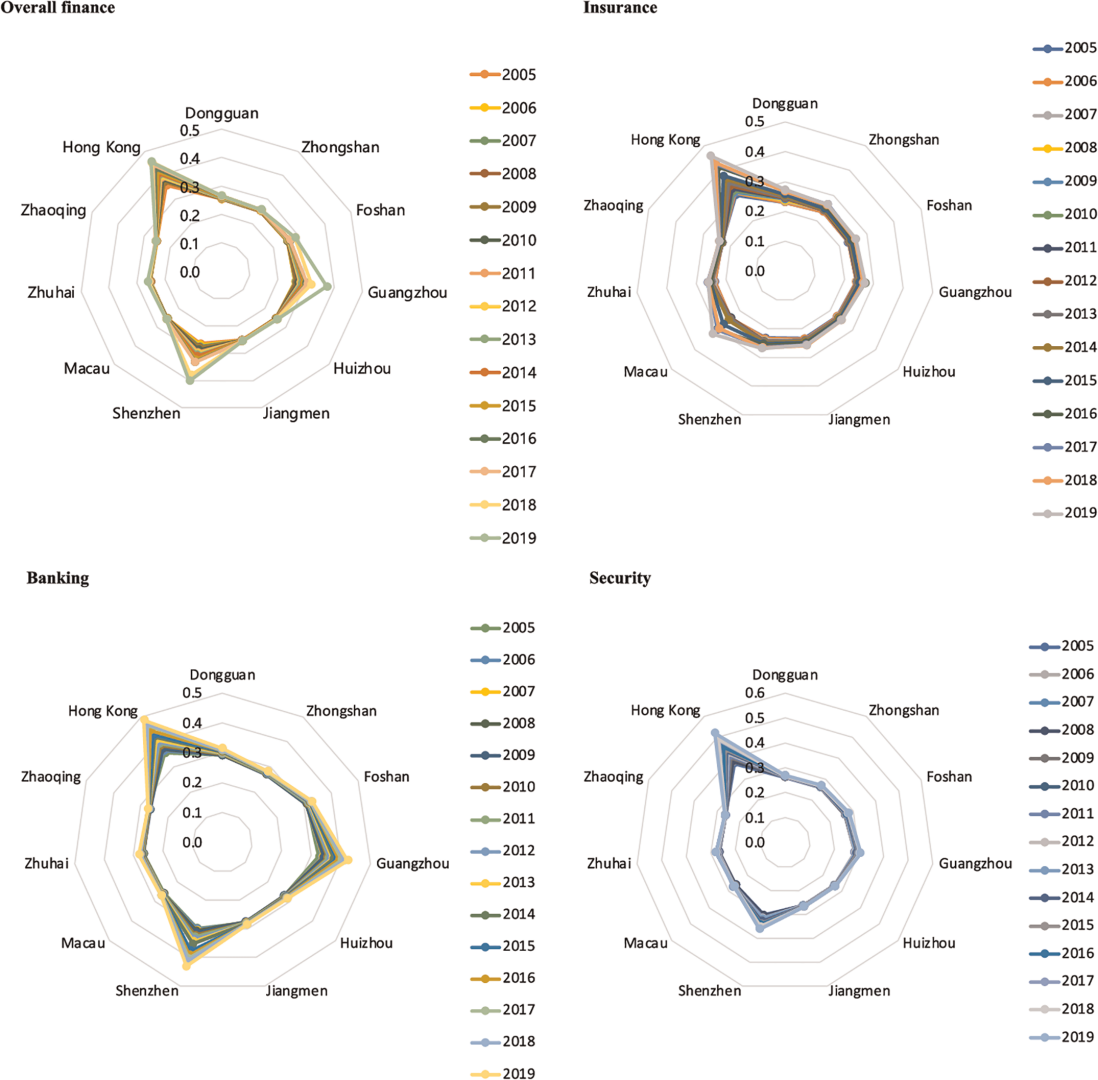
Evaluation of the financial agglomeration of the "9+2" cities in the Guangdong-Hong Kong-Macao Greater Bay Area by dimension (overall financial system, insurance industry, banking industry and securities industry) (2005–2019).

Each city within the "9+2" cities of the GBA has distinct strengths in different financial sectors, including overall financial scale, insurance, banking, and securities (Fig 2). Hong Kong leads in all financial dimensions. In the insurance sector, Hong Kong holds a significant advantage, while Macao shows a relative strength due to its tourism-driven economy. The Mainland cities have niches in specialized sectors, but overall, their insurance agglomeration is less prominent. In the banking sector, Shenzhen and Guangzhou have made significant progress, reducing Hong Kong’s dominance. Zhuhai and Foshan have also seen growth in banking agglomeration. Shenzhen, known for its economic output and financial innovations, and Guangzhou, with its economic strength and market demand, have attracted numerous financial institutions. Zhuhai benefits from regional integration, and Foshan has advanced within the PRD. Hong Kong remains dominant in the securities sector with a sophisticated market infrastructure attracting substantial capital and investor participation. The Shenzhen Stock Exchange is prominent in Mainland China but lacks the international influence and market diversity of Hong Kong. Other cities in the GBA, such as Guangzhou, have less presence in the securities market, though Guangzhou’s robust trading ecosystem supports its banking sector.

### (2) Spatial dynamics of financial agglomeration in the GBA

**Fig 3:**
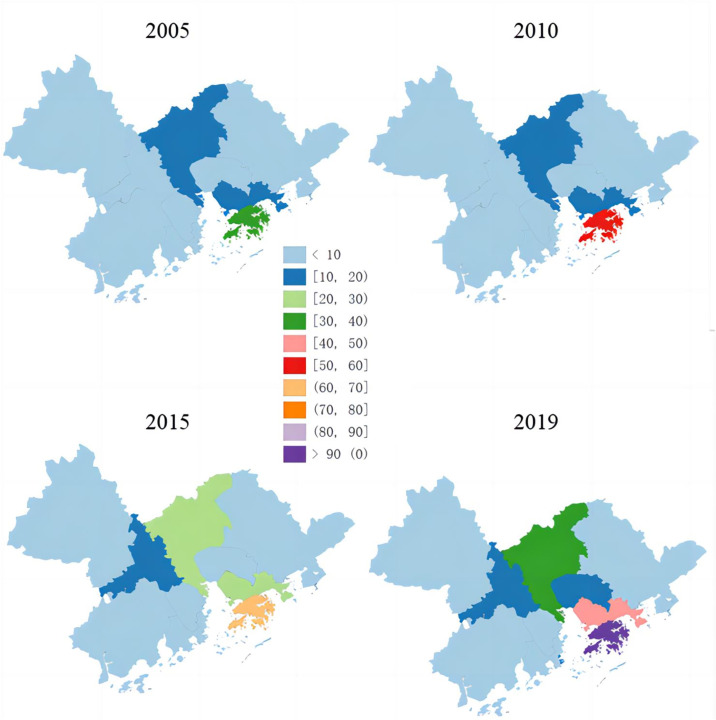
Spatial and temporal map of financial agglomeration in the "9+2" cities in the Guangdong-Hong Kong-Macao Greater Bay Area (2005–2019).

The spatial pattern of financial agglomeration within the GBA presents a complex and dynamic picture. By visualizing agglomeration at different points in time (2005, 2010, 2015, 2019), we outline the changes in financial concentration across the "9+2" cities (Fig 3). The analysis shows a general increase in financial agglomeration scores for most cities within the GBA, with Hong Kong, Shenzhen, and Guangzhou exhibiting the most significant changes. Hong Kong’s financial agglomeration increased from under 40 points in 2005 to over 90 points by 2019. Similarly, Shenzhen’s score rose from under 20 points in 2005 to over 40 in 2019, and Guangzhou’s from under 20 in 2005 to more than 30 in 2019. Cities like Foshan and Dongguan also saw growth, moving from fewer than 10 points in 2005 to over 10 points by 2019. However, cities such as Zhaoqing, Jiangmen, and Huizhou lag behind, remaining in the lower tier of financial development. These disparities in financial agglomeration within the GBA have significant socio-economic implications. While a thriving financial sector can drive economic prosperity, large developmental gaps can lead to social inequality. Uneven distribution of resources and opportunities can widen the gap between rich and poor, potentially destabilizing the region and hindering sustainable growth. This analysis highlights the need for a more balanced approach to regional development, promoting inclusivity and reducing disparities.

When evaluating the spatial dynamics of financial agglomeration across the GBA, findings from Global *Moran’s I* (Table 3) provide insights into the spatial characteristics from 2005 to 2009:

- Significance Assessment: Throughout 2005 to 2009, all recorded p-values were consistently less than 0.1. This indicates that the financial agglomeration data for each year significantly meet the z-value criteria at a 10% significance level, suggesting that the observed spatial pattern is unlikely due to random variation.
- Positive Spatial Autocorrelation: The positive values of Global *Moran’s I* indicate a prevailing positive spatial autocorrelation. This means areas with high financial agglomeration are spatially close to similar areas, showing a clustering effect.
- Evolving Spatial Trends: The upward trend of Global Moran’s I highlights a deepening spatial autocorrelation over the years studied. This suggests that areas with similar financial agglomeration levels are increasingly clustering together.

**Fig 4:**
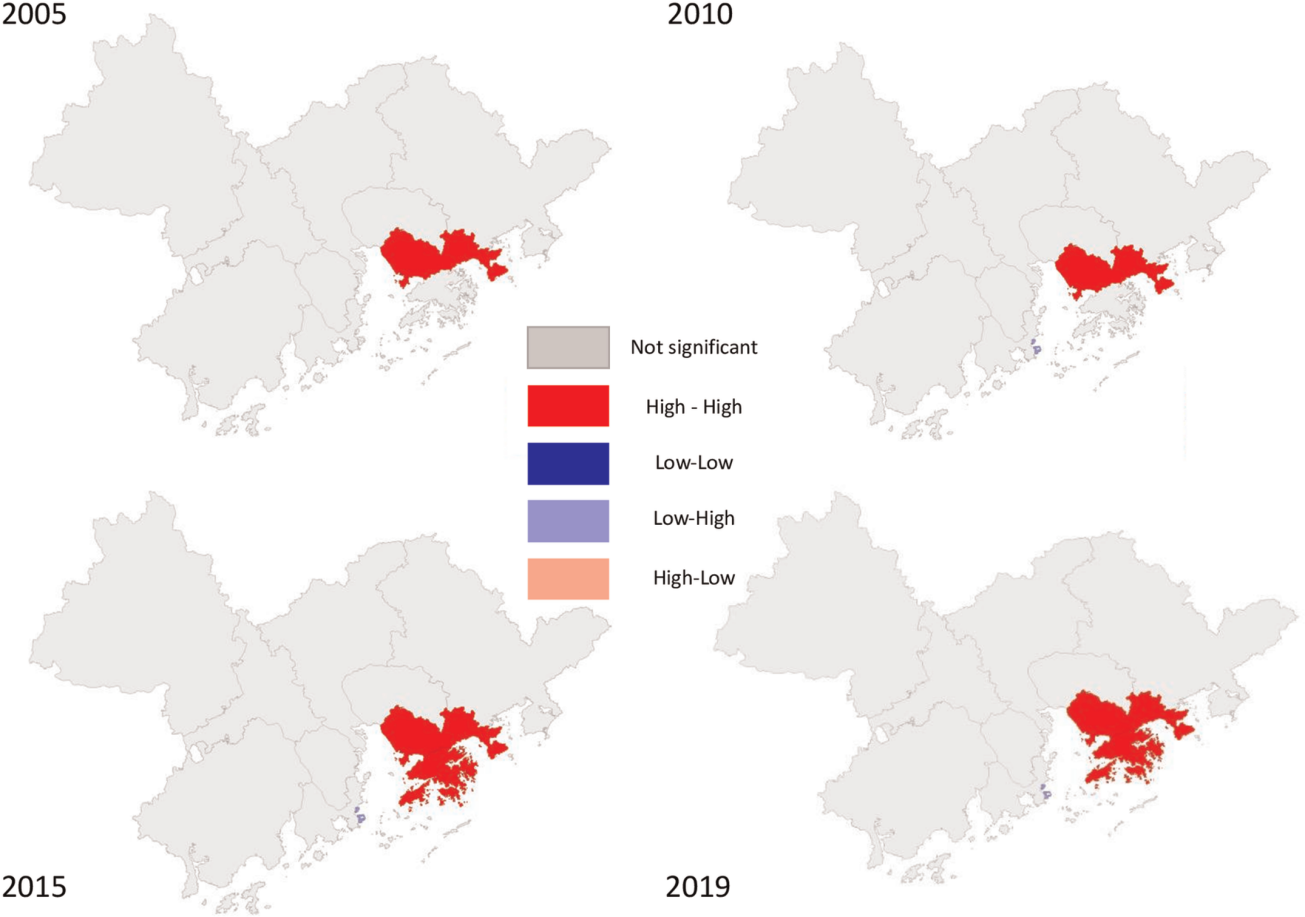
Local Indicators of Spatial Association (LISA) map of the he Guangdong-Hong Kong-Macao Greater Bay Area (2005–2019).

The Local Indicators of Spatial Association (LISA) map of the GBA (Fig 4) reveals significant spatial dynamics. Notably, the urban areas surrounding Shenzhen and Hong Kong form a “high-high” agglomerative cluster. This indicates that cities with high financial concentration, like Hong Kong and Shenzhen, are adjacent to others with similar financial intensity. This clustering effect is driven by the robust financial activities in these cities, attracting resources and talent from neighboring areas, creating a positive feedback loop that amplifies financial agglomeration. Additionally, these leading cities can positively influence the financial landscapes of nearby regions, helping their financial sectors grow. In contrast, post-2010, Macau shows a “low-high” agglomeration pattern. Despite Macau’s slower financial sector growth, its proximity to cities like Zhuhai, which have higher financial concentration, suggests that these neighboring cities benefit from their location near more financially active centers.

**Table 3:** Global Moran Index for the Guangdong-Hong Kong-Macao Greater Bay Area.

Year	Moran’s I	p-value	I	E[I]	mean	sd	z-value
2005	0.05	0.09	0.05	-0.10	-0.10	0.10	1.46
2006	0.07	0.08	0.07	-0.10	-0.10	0.11	1.57
2007	0.09	0.07	0.09	-0.10	-0.10	0.11	1.65
2008	0.07	0.08	0.07	-0.10	-0.10	0.11	1.51
2009	0.10	0.07	0.10	-0.10	-0.10	0.12	1.66
2010	0.11	0.07	0.11	-0.10	-0.10	0.12	1.67
2011	0.11	0.07	0.11	-0.10	-0.10	0.12	1.70
2012	0.12	0.06	0.12	-0.10	-0.10	0.12	1.77
2013	0.13	0.06	0.13	-0.10	-0.10	0.12	1.86
2014	0.14	0.06	0.14	-0.10	-0.10	0.13	1.84
2015	0.18	0.04	0.18	-0.10	-0.10	0.14	2.04
2016	0.21	0.03	0.21	-0.10	-0.10	0.14	2.22
2017	0.20	0.03	0.20	-0.10	-0.10	0.14	2.21
2018	0.21	0.04	0.21	-0.10	-0.10	0.15	2.13
2019	0.25	0.03	0.25	-0.10	-0.10	0.15	2.26

### (3) Determinants influencing financial agglomeration in the GBA

**Fig 5:**
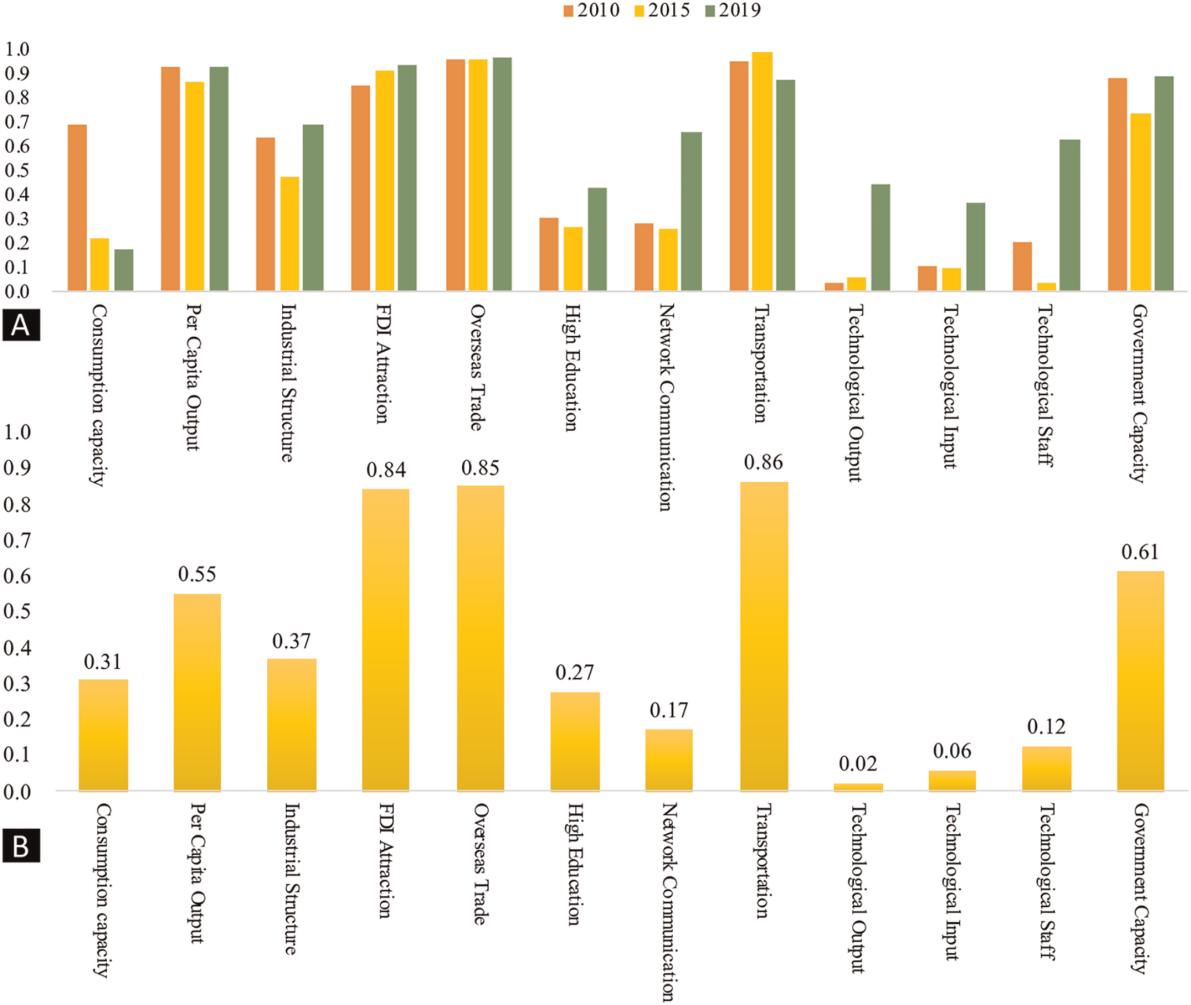
Geo-detection of financial agglomeration influential factors in the Guangdong-Hong Kong-Macao Greater Bay Area. (A) Geo-detection of financial agglomeration influential factors based on time-varying financial agglomeration influential factors (B) Geo-detection of financial agglomeration influential factors based on overall time periods.

Using the GeoDetector, we examined the determinants influencing financial agglomeration in the GBA from 2005 to 2019. Insights from Fig 5B reveal considerable variation in the impact of these determinants over the years. Three primary factors emerged as crucial determinants: transportation infrastructure (q = 0.86), FDI attraction (q = 0.85), and overseas trade (q = 0.84). These align with the region’s tendency towards externally oriented economic development. Other significant variables include governmental capacity (q = 0.61), per capita output (q = 0.55), industrial structure (q = 0.37), and consumption capacity (q = 0.31). In contrast, factors like human capital and technology had relatively less impact on financial agglomeration dynamics.

Over different time periods, the impact of technology-related factors—such as technology inputs, outputs, and specialized human resources—on financial agglomeration has increased significantly (Fig 5A). This trend highlights the growing role of technology within the financial sector. Information technology (IT) and artificial intelligence (AI) have become integral to financial services, influencing payment systems, financial product development, risk management, and customer services. Technologically skilled individuals are highly sought after by financial institutions, forming the core of their recruitment strategies. The GBA, particularly Shenzhen, exemplifies this technological growth. Shenzhen, often compared to Silicon Valley, is a hub of technological innovation, emphasizing the integration of finance and technology.

Similarly, the impact of higher education and network communication on financial agglomeration has also increased. The financial sector’s expanding demand for skilled professionals underscores the importance of higher education institutions. Additionally, the rise of digitalization and widespread internet access have enhanced financial services, benefiting from the GBA’s advanced network infrastructure. Conversely, the influence of consumption power has decreased. Historically, consumer credit and demand drove the financial sector, but now, the focus has shifted towards technology-driven models. The rise of Financial Technology (FinTech) exemplifies this shift, reducing reliance on consumer-centric factors.

**Fig 6:**
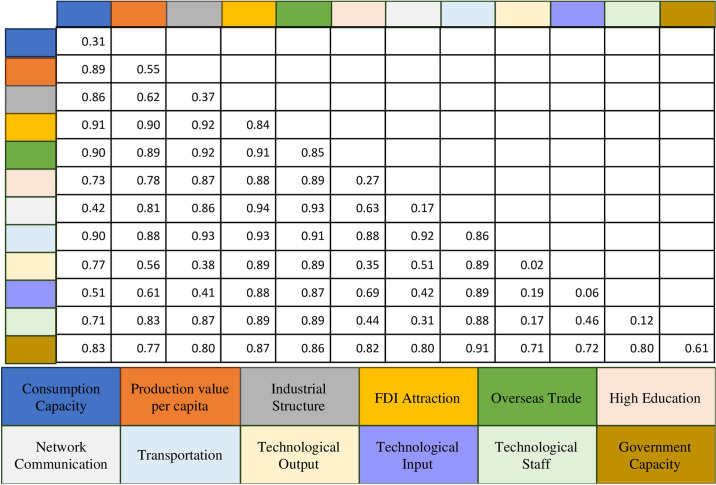
Interactive detection matrix of factors affecting financial agglomeration in the Guangdong-Hong Kong-Macao Greater Bay Area.

Interactive detection analysis confirms that all influencing variables exert an interactive effect on financial agglomeration. The combination of any two factors has a greater impact than each factor individually, manifesting in two types of interactions: nonlinear enhancement and two-factor enhancement (Fig 6). Nonlinear enhancement means the combined explanatory power of interacting factors exceeds their individual effects, indicating complex and synergistic interactions. Two-factor enhancement implies that the presence of two factors together amplifies their impact on financial agglomeration more than their separate effects. Here are three notable examples:

- Consumption Capacity and Technological Output (43.9% increase): A market with high consumption power and a high-tech environment collaboratively fosters financial agglomeration. Elevated consumption attracts financial entities and capital, while technological innovation draws financial investment, promoting financial market growth.
- Industrial Structure and Technological Staff (38.4% increase): The synergy between an optimized industrial structure and an influx of technological personnel boosts financial agglomeration. An advanced industrial structure attracts and retains talented innovators, whose efforts facilitate industrial upgrades and enhancements.
- Higher Education and Technology Input (35.4% increase): The interaction between higher education and technological input creates a virtuous cycle, driving financial agglomeration. Advanced higher education nurtures research talent, elevating technological capabilities. Concurrently, substantial technology investments provide resources and opportunities for higher education, attracting more research talent.

These interactive effects highlight the multifaceted and interdependent nature of the variables influencing financial agglomeration. The findings suggest an integrated approach in policy formulation, considering the synergistic and nonlinear interactions of these factors to develop comprehensive strategies that leverage their collective potential in promoting financial agglomeration in the GBA.

## 4.Discussion

### (1) How far is the future of the three cores?

In recent years, both Shenzhen and Guangzhou have experienced rapid development in the financial industry, gradually catching up to Hong Kong in terms of overall sector scale. The banking sector in these cities has shown significant growth. However, Hong Kong maintains a clear advantage in securities and insurance. Hong Kong remains the primary financial center of the GBA, while the development of Shenzhen and Guangzhou has created a three-core structure. However, significant variations in financial development exist within the GBA. For instance, Hong Kong, Shenzhen, and Guangzhou have financial outputs exceeding 100 billion yuan, while cities like Zhaoqing have outputs below 10 billion yuan. Hong Kong has the highest overall financial development level, although some studies suggest that the mainland Chinese financial industry surpasses that of Hong Kong [35]. Moreover, Hong Kong’s securities and insurance sectors maintain a significant scale advantage and serve as an international asset and risk management center in the GBA [36].

Since the 1970s, Hong Kong has grown rapidly in the banking industry, becoming one of the world’s top four banking centers and a crucial financial hub [37]. It attracts substantial foreign investment in the banking sector more effectively than other Chinese cities. While mainland cities may not have an absolute advantage in banking, they have a comparative advantage in other dimensions. Hong Kong’s dominance in banking agglomeration has slightly diminished, with Shenzhen and Guangzhou catching up. Guangzhou shows a comparative advantage in banking, and the banking sectors in Zhuhai and Foshan are also improving rapidly. Unlike the typical polarization effect in the finance industry, each city in the GBA maintains a high banking penetration rate, with even economically underdeveloped cities matching their local economic scale in banking resources.

The Hong Kong Stock Exchange and Shenzhen Stock Exchange have developed significant advantages in the securities industry within the GBA. Both cities possess specialized strengths in the multi-level capital market system within the GBA and nationwide [38]. These exchanges facilitate the concentration of securities firms, bond registration, and rating agencies, providing superior financial products and services such as stocks, bonds, and futures for local companies [39]. The establishment of the Shanghai Stock Exchange South Center in Guangzhou on September 10, 2019, offers new opportunities for the development of the securities industry in Guangzhou.

Hong Kong is a major hub for insurance in the Asia-Pacific region and is recognized as having one of the world’s premier insurance sectors. Over half of the top global insurance companies have business units in Hong Kong [40], and many international insurance companies choose Hong Kong as their regional headquarters [41]. These factors have established Hong Kong as an insurance stronghold. With more advanced, competitive, and affordable insurance products compared to the nine PRD cities [42], Hong Kong attracts many mainland tourists seeking insurance coverage. The competition among numerous insurance companies in Hong Kong results in higher-quality services at more affordable prices compared to the Mainland.

The dynamic nature of financial markets, coupled with strategic decisions by financial institutions, leads to changes in the size and configuration of financial agglomeration over time and space [43]. This differentiation trend can be attributed to variations in the maturity of the GBA’s financial markets and levels of economic development. Policymakers need to consider inter-regional variations and formulate specialized policies to promote the growth of the financial industry.

### (2) Is the spatial agglomeration of finance the Matthew effect?

The Matthew effect is evident in the spatial concentration of finance, as demonstrated by Zeng et al. [44]. The Matthew Effect suggests that entities already endowed with substantial resources tend to accumulate even more, thereby enhancing their advantage. This is similar to the adage, "the rich get richer" [45]. In the financial sector, this effect is clear, as financial institutions and services tend to cluster in specific locations, creating financial hubs. In the GBA, this trend of financial agglomeration is noticeable. Cities with significant financial prominence, such as Hong Kong and Shenzhen, mutually reinforce and sustain each other’s growth, forming a "high-high" clustering pattern. These cities attract financial institutions and capital due to their advanced financial infrastructure, further consolidating their financial status.

The financial sector relies heavily on information and trust. Financial agglomerations serve as crucial hubs, enhancing accessibility and information sharing among financial entities. Such concentration improves efficiencies by reducing transaction costs [46]. Additionally, the clustering of financial activities fosters innovation. The proximity of financial institutions creates an environment conducive to both competition and cooperation, leading to the development of new financial tools and services [47]. This dynamic stimulates innovation, driving the finance industry to new levels of growth and sophistication.

Over the past four decades since the inception of the Shenzhen Special Economic Zone, the collaboration between Shenzhen and Hong Kong has expanded, marked by continuous innovations in their cooperative framework. This evolution includes the "Three Plus One" (*San Lai Yi Bu*) trade mix, the "Twin-city Economy", and the integration into the "GBA" framework [48]. Central to this collaboration is the financial sector. Shenzhen has adopted advanced practices from Hong Kong’s financial sector, enhancing financial market collaboration, cross-border financial engagements, and innovation in financial products [49]. A prime example is the "Shenzhen-Hong Kong Stock Connect", facilitating economic exchanges and mobile payments between residents of Shenzhen and Hong Kong. This integration has led to a marked increase in financial agglomeration between the two cities, exhibiting a "high-high" correlation.

### (3) Which is the promising power in financial development?

The intricacies of financial agglomeration within the GBA can be understood through the Geodetector. Key drivers include transportation, FDI attraction, and overseas trade. An efficient transportation system is crucial, reducing transaction costs and facilitating agglomeration [50]. The ability to attract foreign investment reflects a region’s openness and inclusiveness, drawing multinational financial institutions that bring fintech innovations, international business practices, and capital. Overseas trade also plays a significant role in financial agglomeration. Increasing global trade boosts demand for financial services, driving innovation. A strong trade mechanism increases the need for trade finance, prompting the growth of financial entities, including banks and insurers.

The influence of technological determinants has grown significantly. Modern financial geography emphasizes the role of technology in financial agglomeration [51]. Financial technology (FinTech) has enhanced the operational efficiency of financial services, improving transaction fluidity and accessibility [52]. Shenzhen, home to tech giants like Tencent and Huawei, exemplifies this confluence of finance and technology. Technological advancements drive the development of new financial instruments, attracting institutional and investor interest, and promoting financial agglomeration. Innovations like blockchain technology support decentralized finance (DeFi), while AI and machine learning improve risk assessment and investment strategies.

The relationship between innovation and technology is crucial for the sustainability of financial agglomeration. Financial centers, as knowledge-intensive sectors, benefit from cluster learning dynamics and innovation ecosystems [37]. Financial entities within these agglomerations create innovation networks that foster shared knowledge and continuous improvement [53]. In contrast, the influence of the consumption factor has declined. This shift is linked to the rise of financial technology and the globalization of financial markets. FinTech has increased efficiency, allowing consumers to access financial services regardless of location. Globalization has facilitated the movement of capital, labor, and knowledge, reducing the importance of local consumer demand. Financial institutions are focusing more on optimizing returns and risk management for corporate and institutional clients rather than individual consumers.

### (4) Focus on financial development in the GBA in the future

The Geodetector model identified two primary interaction types impacting financial agglomeration—nonlinear enhancement and two-factor enhancement—which produce synergistic effects. For example, combining consumption capacity and technical output increases demand for innovative products and drives technological innovation in financial institutions. The interaction between industrial structure and technical staff stimulates industrial improvement and innovation, strengthening local financial sectors. Additionally, the synergy between higher education and technical input boosts regional innovation capabilities and improves the quality of education and talent in science and technology (S&T) fields.

There is a symbiotic relationship between consumption capacity and technological innovation, which is crucial for financial agglomeration. For the GBA, harmonizing consumption capacity with scientific and technological advancement is essential. Technological innovations like e-commerce, Internet banking, and electronic payments have increased consumption levels and diversified consumption methods, boosting demand. Enhanced consumption supports the commercialization of scientific and technological innovations, attracting financial resources. Governments should invest in R&D and elevate consumption by increasing public incomes, expanding social security, and enhancing education and training. Strengthening this interaction will spur financial sector growth.

The GBA should also emphasize fostering scientific innovation, focusing on developing talent exchange and collaboration networks. Industrial refinement requires technical personnel, who thrive in a robust industrial environment. This relationship strengthens the region’s economic appeal. A sophisticated industrial structure relies on technological innovation, driven by skilled personnel. Their interaction guides the industry towards high-end development, expanding the market for the financial sector and promoting financial agglomeration. Concentrating S&T talents in areas like Shenzhen/Hong Kong and Zhuhai/Macao will maximize their potential. The GBA should maintain its competitive edge in strategic industries like biomedicine, new materials, and semiconductors, and support advanced manufacturing to become a global S&T innovation hub.

Higher education institutions play a crucial role in cultivating and attracting talent, supporting scientific research, and building talent reservoirs. Technical input fosters innovation, attracting skilled individuals. This dynamic interaction accelerates financial innovation, drawing more financial institutions and capital. Higher education provides the workforce necessary for S&T and financial sector innovation. These professionals, with their knowledge and innovative thinking, drive financial product and service innovation. Technical input supports this innovation, leading to technological advancements that enhance the financial sector’s efficiency and capacity. For sustained development, the GBA must focus on higher education, fostering collaboration among cities like Shenzhen, Guangzhou, Hong Kong, and Macau, and leveraging university partnerships to improve educational resources and standards. The GBA’s strategic goal should be to attract and retain talent in S&T, finance, and related fields, creating a strong talent pool.

### (5) Future research directions: Exploring the dynamics of financial practices and investor behavior in the GBA

By adopting financial practices that avoid excessive speculation and emphasize real economic activities, the GBA could enhance its financial stability. Policies supporting venture funds based on equitable risk and reward distribution, such as those in Islamic banking [54], could foster collaborative financial practices. This approach encourages financial institutions to develop products yielding returns based on actual profits rather than fixed interest rates, aligning with transparent and risk-aware financial management. Such a model, tying credit expansion more directly to real asset and service growth, offers a way to mitigate systemic risks inherent in conventional credit systems.

Studies by Aydilek [55, 56] show how habit formation and the allocation of non-market time influence household decisions, potentially impacted by financial agglomeration. Understanding these behaviors within dense financial environments offers insights into economic modeling in urban settings. Research by Coval and Moskowitz [57]and Seaholes and Zhu [58] demonstrates how geographical and professional proximities shape investor behavior, highlighting the significant effects of financial agglomeration on investment choices. These findings suggest that financial clusters may induce localized biases in investment decisions, crucial for understanding market dynamics within the GBA.

Building on these studies, future research should investigate the impact of financial agglomeration on household decision-making in the GBA. Such studies would explore how proximity to major financial centers influences household financial behaviors and decisions, offering valuable insights for policymakers and financial planners. This research direction promises to enrich our understanding of the interplay between financial infrastructure and economic behavior, facilitating more informed and effective policy interventions.

## 5.Conclusion

The financial agglomeration levels within the the GBA exhibit a consistent upward trajectory, revealing not only growth but also an expanding disparity among cities. This phenomenon is significantly influenced by factors such as transportation, overseas trade, and foreign investment, which are integral to the GBA’s export-oriented economic structure. Notably, the increasing impact of technology on financial agglomeration mirrors the rise of FinTech, whereas the influence of consumption capacity shows a decline. Furthermore, the research identifies complex interactions among these determinants, with dual-factor impacts generally more pronounced than those of single factors due to nonlinear enhancements and synergistic effects. This study underscores the intricate and evolving dynamics of financial agglomeration in the GBA. The integration of principles from Islamic banking suggests alternative pathways to enhance financial stability and ethical practices within the region. This approach, emphasizing real economic activities and equitable risk distribution, provides a viable model to mitigate systemic risks and align financial practices with sustainable and transparent management. Future research should further explore how financial agglomeration influences household decision-making and investment behaviors, particularly in the context of the growing financial centers in the GBA. Investigating these relationships will yield deeper insights into the regional economic framework, offering valuable guidance for policymakers and financial planners aiming to foster a resilient and inclusive financial ecosystem.

## Supporting information

S1 File (ZIP)
