# Supplementary material for: Spatial patterns and influencing factors of financial agglomeration in Guangdong-Hong Kong-Macao Greater Bay Area
Source: PLoS One. 2024 Aug 1;19(8):e0306301. doi: 10.1371/journal.pone.0306301 (PMC11293679; doi:10.1371/journal.pone.0306301)
Supplement: S1 File — (ZIP) [file pone.0306301.s001.zip › S1 Text.docx]

**S1 Text: Detailed computational steps for the entropy weight method used in evaluating the agglomeration degree of the financial industry.**

Detailed computational steps for the entropy weight method used in evaluating the agglomeration degree of the financial industry.

we employ the entropy weight method for weight assignment in evaluating the agglomeration degree of the financial industry. The computational steps for the entropy weight method are delineated as follows:

Weight of indicator *j* in region *i*：

$$P_{ij}=\frac{X_{ij}}{\sum_{i=1}^{n} X_{ij}}$$

Information entropy of indicator *j*

$$E_{ij}=-\frac{1}{\ln n}\sum_{i=1}^{n} (p_{ij}\times\ln P_{ij})$$

Utility value of indicator *j*：

$$D_{j}=1-E_{j}$$

Weight of indicator *j*：

$$W_{j}=\frac{D_{j}}{\sum_{j=1}^{m} D_{j}}$$

*n* denotes the number of cities and *m* denotes the number of indicators.

The composite score of the indicator for the city *i* is obtained based on the weights, with the following equation：

$$S_{i}=\sum_{j=1}^{m} W_{i}\times X_{ij}$$
