# Supplementary material for: Spatial patterns and influencing factors of financial agglomeration in Guangdong-Hong Kong-Macao Greater Bay Area
Source: PLoS One. 2024 Aug 1;19(8):e0306301. doi: 10.1371/journal.pone.0306301 (PMC11293679; doi:10.1371/journal.pone.0306301)
Supplement: S1 File — (ZIP) [file pone.0306301.s001.zip › S2 Text.docx]

**S2 Text: Formulas and principles of Spatial Auto Correlation and Local Moran's I metrics used to assess the spatial pattern of financial agglomeration within the GBA.**

To assess the spatial pattern of financial agglomeration within the GBA, we utilize Spatial Auto Correlation as our primary metric to measure spatial relevance.

The computational formulae for these indices are presented below:

$I=\frac{n\sum_{i=1}^{n} \sum_{j=1}^{n} w_{ij}\left( x_{i}-\bar{x} \right)(x_{j}-\bar{x})}{{\sum_{i=1}^{n} \left( x_{i}-\bar{x} \right)}^{2}\sum_{j=1}^{n} w_{ij}}$ ,$W=\left[ \begin{matrix} \begin{matrix} w_{11} & w_{12} \\ w_{21} & w_{22} \end{matrix} & \cdots& \begin{matrix} w_{1n} \\ w_{2n} \end{matrix} \\ \vdots& \ddots& \vdots\\ \begin{matrix} w_{n1} & w_{n2} \end{matrix} & \cdots& w_{nn} \end{matrix} \right]$

$x_{i}$ is the attribute value of variable $x$ at spatial location $i$, $\bar{x}$ is the mean value of the variable; $n$ denotes the total number of different regions in different years; $w_{ij}$is the spatial weight matrix, which refers to the spatial weight of location *i* and *j*, and contains a variety of connectivity, such as geographic connectivity, economic connectivity, and so on. The principles of the spatial autocorrelation test are based on the calculation of the standard deviation Z of the approximate standard normal distribution. If *Z* is greater than 0, it indicates a positive association; if *Z* is less than 0, it indicates a negative association.

$Moran^{'}I>0$ indicates that there is an overall positive spatial correlation, the closer the correlation, the larger the value, the more obvious the spatial correlation, which implies that areas with similar attributes tend to cluster; $Moran^{'}I<0$indicates that there is an overall negative spatial correlation, the smaller the value, the larger the spatial difference, and the areas of the opposite attributes tend to cluster; and $Moran^{'}I=0$, which indicates that there is no correlation, and the space is stochastic.

To further refine the spatial analysis of financial agglomeration in the GBA, we have incorporated the local $Moran^{'}I_{i}$, which reflects the spatial correlation of financial agglomeration between region i and its neighboring regions. The local correlation test of $Moran^{'}I_{i}$, also known as the LISA metric, is a measure of spatial autocorrelation that is commonly used. The formula is calculated as follows:

$I_{i}=z_{i}\sum_{j=1}^{n} w_{ij}z_{ij}$ ,

$$W=\left[ \begin{matrix} \begin{matrix} w_{11} & w_{12} \\ w_{21} & w_{22} \end{matrix} & \cdots& \begin{matrix} w_{1n} \\ w_{2n} \end{matrix} \\ \vdots& \ddots& \vdots\\ \begin{matrix} w_{n1} & w_{n2} \end{matrix} & \cdots& w_{nn} \end{matrix} \right]$$

$z_{i}$ and $z_{j}$are the normalised attribute values of $x_{i}$and $x_{j}$for neighbouring spaces *i* and *j*, respectively.

$z_{i}=\frac{x_{i}-\bar{x}}{\sigma_{x}}$，$z_{j}=\frac{x_{j}-\bar{x}}{\sigma_{x}}$，$\sigma_{x}=\sqrt{\sum_{i=1}^{n} {a(x_{i}-\bar{x})}^{2}/N}$

$I_{i}$ is the calculated value of Moran of observation unit $i$.

Similarly, for the local Moran index, $Z\left（ I_{i} \right）>0$ denotes a consistent agglomeration feature; $Z\left（ I_{i} \right）<0$ denotes a divergent agglomeration feature.
