# Supplementary material for: Spatial patterns and influencing factors of financial agglomeration in Guangdong-Hong Kong-Macao Greater Bay Area
Source: PLoS One. 2024 Aug 1;19(8):e0306301. doi: 10.1371/journal.pone.0306301 (PMC11293679; doi:10.1371/journal.pone.0306301)
Supplement: S1 File — (ZIP) [file pone.0306301.s001.zip › S3 Text.docx]

**S3 Text: Data on influential factors of financial agglomeration in the GBA, including consumption capacity, per capita output, industrial structure, FDI attraction, overseas trade, high education, network communication, transportation, technological output, technological input, technological personnel, and government capacity.**

Influential Factors of Financial Agglomeration in the GBA are as follows:

- Consumption Capacity: Reflects the purchasing power and spending trends of the population.
- Per Capita Output: Measures the average economic productivity per individual, signifying the economic vitality of the area.
- Industrial Structure: Assesses the composition and interrelation of various industries and their influence on financial agglomeration.
- FDI Attraction: Evaluates the region's ability to draw international capital and investments.
- Overseas Trade: Analyses the scale and scope of the region's international trade activities and their impact on financial activities.
- High Education: Focuses on the role of higher education, considering its outputs for talent development.
- Network Communication: Probes into the digital and telecommunications infrastructure, emphasizing its role in modern financial systems.
- Transportation: Studies the efficiency and connectivity of the transportation network, critical for facilitating economic and financial activities.
- Technological Output: Measures the region's innovation outputs, such as patents, which can spur financial activities.
- Technological Input: Gauges the level of investment in research and development, signifying commitment to innovation.
- Technological Personnel: Evaluates the presence of professionals in science and tech sectors, highlighting the human capital aspect.
- Government Capacity: Considers the influence of government in promoting or inhibiting financial agglomeration.
