# Supplementary material for: Spatial patterns and influencing factors of financial agglomeration in Guangdong-Hong Kong-Macao Greater Bay Area
Source: PLoS One. 2024 Aug 1;19(8):e0306301. doi: 10.1371/journal.pone.0306301 (PMC11293679; doi:10.1371/journal.pone.0306301)
Supplement: S1 File — (ZIP) [file pone.0306301.s001.zip › S4 Text.docx]

**S4 Text: Detailed steps for GeoDetector analysis, including divergence and factor detection, and interaction detection.**

The specific steps in GeoDetector are as follows:

*Divergence and Factor Detection*: Assess the spatial variation or imbalance of financial agglomeration, denoted as *Y*, as well as understand the influence of various factors (economy, manpower, infrastructure, etc.), denoted as *X*. A higher *q* value signifies a pronounced spatial imbalance in *Y*. If the high *q* value is associated with a particular factor *X*, it indicates that the factor strongly explains or influences the observed imbalance in *Y*.

$$q=1-\frac{\sum_{h=1}^{L} N_{h}\sigma_{h}^{2}}{N\sigma^{2}}=1-\frac{SSW}{SST}$$

$$SSW=\sum_{h=1}^{L} N_{h}\sigma_{h}^{2},SST=N\sigma^{2}$$

$h=1, \ldots, L$ represents each study subregion; $N_{h}$and $N$ represent the number of units in different partitions; $\sigma_{h}^{2}$is the variance of the subregion h, and $\sigma^{2}$is the variance of the whole region of financial agglomeration *Y*. $SSW$ is the sum of the variances of the different subregions, and SST is the total variance of the whole study region. The range of the detection value *q* is [0,1], the larger the value of *q* is, the more obvious the spatial imbalance of financial agglomeration *Y*.

*Interaction Detection*：When studying the impact of different influencing factors X (economic, human, and infrastructure variables) on a particular outcome, such as financial agglomeration, it's not just individual effects that are important but also their potential interactions. These interactions can amplify or diminish the effects that individual factors have when acting alone. The method of assessment is to first calculate the $q$ value, $q(X_{1})$ and $q(X_{2})$，of the two impact factors $X_{1}$ and $X_{2}$on the financial agglomeration *Y*, respectively, and calculate the interaction $q \mathrm{value}$, $q\left( X_{1}\cap X_{2} \right)$, then compare $q(X_{1})$ and $q(X_{2})$ with $q\left( X_{1}\cap X_{2} \right)$.

When $q\left( X_{1}\cap X_{2} \right)$ is smaller than the smallest one of $q(X_{1})$ and $q(X_{2})$, the explanatory power of $X_{1}$ and $X_{2}$ on financial agglomeration Y has a nonlinear weakening relationship.

when $q\left( X_{1}\cap X_{2} \right)$is larger than the smallest one of $q(X_{1})$ and $q(X_{2})$，and smaller than the largest one of them both, the explanatory power of $X_{1}$ and $X_{2}$ on financial agglomeration *Y* has a one-way nonlinear weakening relationship;

when $q\left( X_{1}\cap X_{2} \right)$ is greater than the largest of $q(X_{1})$ and $q(X_{2})$ , the explanatory power of $X_{1}$ and $X_{2}$ on financial agglomeration *Y* has a two-factor enhancing relationship.

when $q\left( X_{1}\cap X_{2} \right)$ is equal to the sum of $q(X_{1})$ and $q(X_{2})$, the explanatory power of $X_{1}$ and $X_{2}$ on financial agglomeration *Y* is mutually independent. shiming

when $q\left( X_{1}\cap X_{2} \right)$ is greater than the sum of $q(X_{1})$ and $q(X_{2})$, the explanatory power of $X_{1}$ and $X_{2}$on financial agglomeration *Y* is in a non-linearly enhancing relationship.
